# Anti-TIF1gamma Antibody-Positive Dermatomyositis Associated with Myelodysplastic Syndrome: Response to Treatment

**DOI:** 10.7759/cureus.5775

**Published:** 2019-09-26

**Authors:** Irina Lerman, Christopher T Richardson

**Affiliations:** 1 Dermatology, University of Rochester School of Medicine and Dentistry, Rochester, USA

**Keywords:** dermatomyositis, myelodysplastic syndrome

## Abstract

Dermatomyositis (DM) classically presents as a dyad of typical cutaneous findings and varying degrees of proximal muscle weakness. Interestingly, the development of DM may signal underlying malignancy, and numerous myositis-specific autoantibodies have been associated with this paraneoplastic phenomenon. Positivity for anti-TIF1gamma antibody, in particular, raises suspicion for cancer-associated DM. Here, we present an unusual case of anti-TIF1gamma antibody-positive DM that ultimately lead to the diagnosis of myelodysplastic syndrome (MDS). Importantly, topical treatment and chemotherapy targeting MDS resulted in a swift and remarkable amelioration of cutaneous disease.

## Introduction

Dermatomyositis (DM) is an idiopathic inflammatory myopathy that classically presents with symmetric proximal muscle weakness accompanied or preceded by signature cutaneous findings such as heliotrope eruption, photo-distributed poikiloderma, and Gottron’s papules. However, the extent of myopathy is variable, with up to 50 percent of DM patients experiencing no to mild muscle weakness and myalgias - termed as clinically amyopathic DM and hypomyopathic DM, respectively [1,2]. Numerous myositis-specific autoantibodies have been recognized to correlate with certain clinical and prognostic features. In particular, an antibody directed against a 155kDa protein, later identified as TIF1gamma, is found in up to 80 percent of cancer-associated DM cases and therefore highly predictive of malignancy [3]. 

Here, we describe a patient with anti-TIF1gamma antibody-positive, initially clinically amyopathic DM who was subsequently diagnosed with myelodysplastic syndrome (MDS). He later developed unilateral myalgia which prompted further workup, revealing radiographic evidence of mild bilateral myositis. To our knowledge, this type of unique presentation of anti-TIF1gamma antibody-positive DM secondary to MDS has not been previously reported. Importantly, chemotherapy for MDS resulted in significant clinical and symptomatic improvement of cutaneous disease. This case further emphasizes the importance of identifying and treating rare underlying malignancies in patients presenting with DM and anti-TIF1gamma antibody positivity.

## Case presentation

A 70-year-old man with a past medical history of diabetes mellitus, benign prostatic hyperplasia, and osteoarthritis status-post recent right total hip arthroplasty initially presented to his primary care physician with an intensely pruritic, scaly, and erythematous scalp. He was unsuccessfully treated with tar shampoo and ketoconazole cream. Over the following month, his rash progressed to involve his eyelids, face, and chest, and he was referred to a dermatologist who prescribed prednisone 60 mg daily and topical triamcinolone 0.1% compounded with phenol and menthol 0.5%. He developed manic symptoms, and prednisone was slowly tapered with resultant improvement in mood. Unfortunately, his rash further progressed to involve the upper back and arms; due to characteristic appearance and pattern distribution of the eruption, DM was suspected. Of note, he denied muscle weakness at this time. 

Skin biopsy of the chest demonstrated focal epidermal atrophy with basal layer vacuolization, rare individual necrotic keratinocytes, mild superficial dermal edema, mild superficial perivascular lymphocytic infiltrate, dilated vessels, and rare extravasated erythrocytes - changes suggestive of interface dermatitis compatible with DM. Direct immunofluorescence revealed cytoid bodies positive for IgG, IgM, IgA, C3, shaggy fibrin deposits, and granular deposits of C5b-C9 at the dermo-epidermal (DE) junction. While somewhat non-specific, these changes were suggestive of a lichenoid process compatible with DM. Initial laboratory studies included negative antinuclear antibody (ANA), normal c-reactive protein (CRP) and erythrocyte sedimentation rate (ESR), normal creatine kinase (CK) and aldolase, and negative anti-Mi2 antibody. Complete blood count revealed leukopenia, anemia, and thrombocytopenia. Hematologic malignancy was suspected, and the patient was referred to an oncologist for further investigation. 

**Figure 1:**
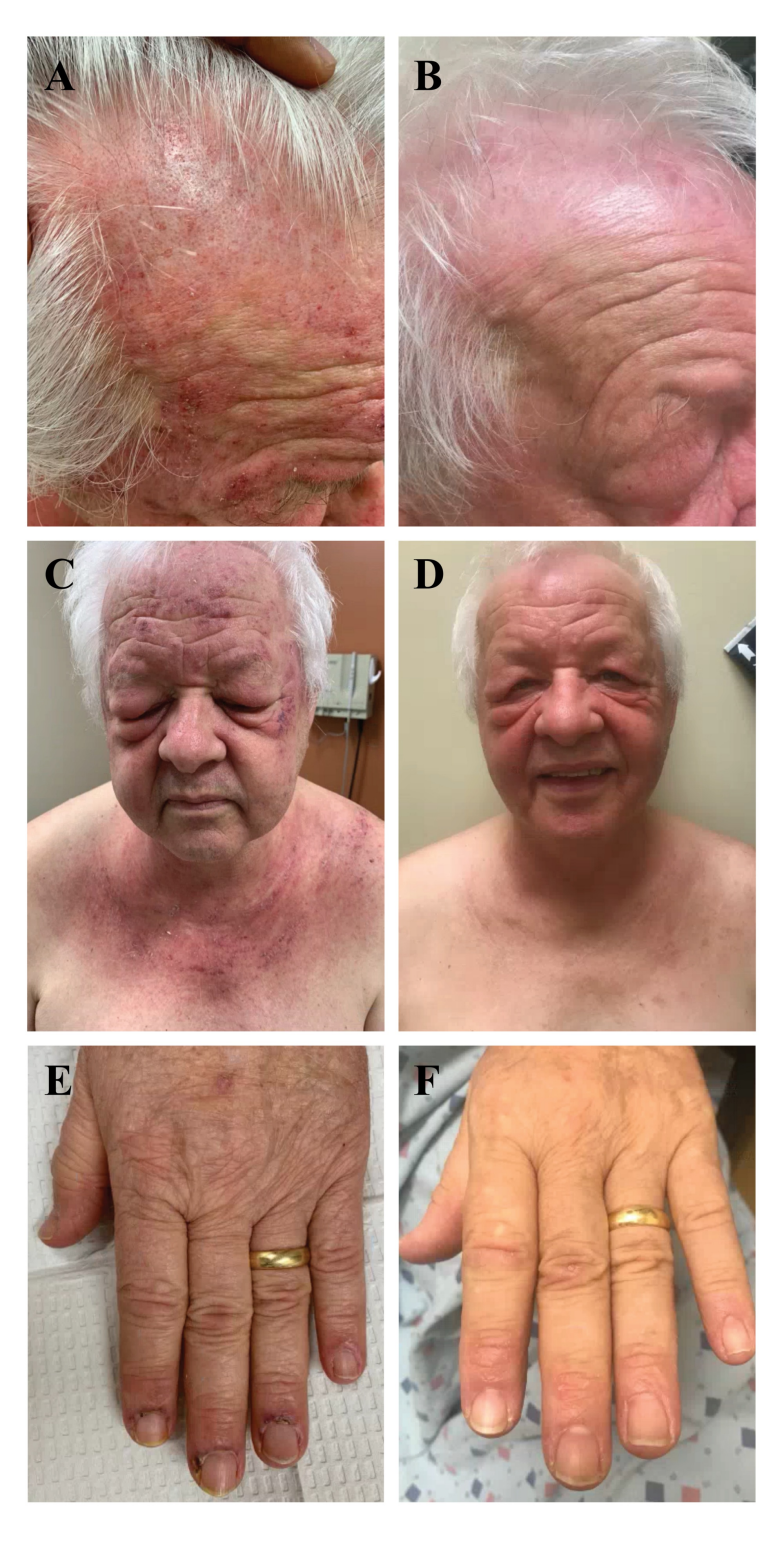
Dermatologic Exam The patient presented with cutaneous features of dermatomyositis, including red-on-white poikiloderma of the scalp (A), violaceous erythema overlying upper eyelids (heliotrope rash), periorbital edema, photo-distributed poikiloderma with violaceous plaques (C), and periungual erythema and tenderness (E). Ten weeks after initiation of chemotherapy for myelodysplastic syndrome, along with only topical treatment, cutaneous symptoms of dermatomyositis were significantly improved (B, D, and F).

At this point, he was also referred to us for additional workup and management of rapidly progressive and likely cancer-associated clinically amyopathic DM. Our exam was significant for red-on-white poikiloderma of the scalp (Figure 1A) and violaceous erythema overlying upper eyelids (heliotrope rash) with significant periorbital edema (Figure 1C). Violaceous psoriasiform papules and plaques with focal areas of purpura were noted on the face, chest (V-sign), upper back (shawl sign), and arms (Figure 1C). On the bilateral hands, there were ragged cuticles and significant periungual erythema, purpura, and tenderness (Figure 1E). Gottron's papules were absent, although faint erythema overlying the distal interphalangeal joints was appreciated (Figure 1E). Again, the patient denied muscle weakness and myalgia. Of note, the patient provided informed consent for publication of the photographs.

Myositis antibody panel testing was pursued and revealed positivity for anti-p155 antibody, consistent with TIF1gamma as previously reported [3]. Meanwhile, bone marrow biopsy performed by the oncology team confirmed MDS with excess blasts (EB-2). Chemotherapy consisting of azacytidine and pevonedistat was initiated per clinical trial protocol. Given that his chemotherapy protocol excluded several systemic therapies, treatment of his MDS was considered most important, and his DM findings were limited to the skin, only topical triamcinolone 0.1% and tacrolimus 0.1% ointment were initiated. Ten weeks into chemotherapy and topical treatment, his rash was noted to be significantly improved, with the resolution of pruritus and no new areas of involvement (Figures 1B, 1D, and 1F). 

**Figure 2:**
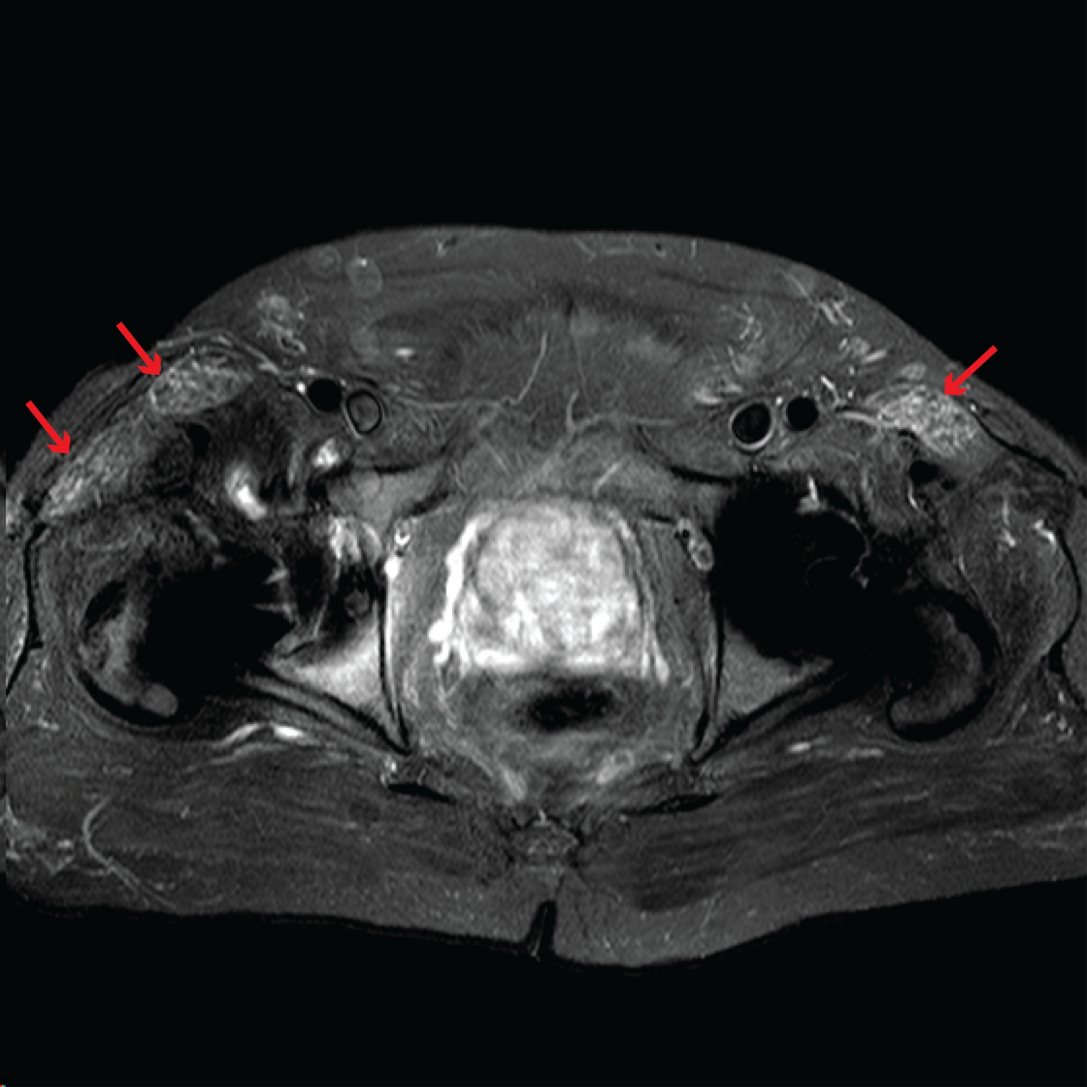
Magnetic Resonance Imaging (MRI) Pelvic MRI showed mild increased short TI inversion recovery (STIR) signal in anterior thigh muscles bilaterally, indicated by red arrows. Findings were consistent with inflammatory myopathy.

In the interim, the patient developed new-onset right-sided hip pain; left lower extremity was unaffected. On manual muscle testing, right hip flexion against resistance elicited pain, but strength was only mildly decreased (grade 4/5), likely due to the pain. Left hip flexion and bilateral shoulder abduction were fully intact (grade 5/5). Repeat laboratory testing revealed normal CK and mildly elevated aldolase of 10.7 U/L (reference range: 1.5-8.1 U/L). Magnetic resonance imaging (MRI) of the pelvis showed mild increased short TI inversion recovery (STIR) signal at the bilateral iliopsoas muscles, bilateral sartorius muscles, and right tensor fasciae latae muscle (Figure 2). These radiographic findings suggested a component of myopathy not previously appreciated in this patient and were consistent with dermatomyositis, which commonly affects the anterior thigh muscles and leads to symmetric proximal muscle weakness [4]. However, it would be unusual for mild bilateral myositis to be the cause of unilateral hip pain, especially given that the typical manifestation of myositis in DM is bilateral weakness; we suspect his hip pain is related to his prior right hip arthroplasty. Due to prior intolerance of systemic steroids (mania), incompatibility of steroid-sparing immunosuppressants with ongoing chemotherapy protocol, relatively mild muscle symptoms, and overall significant improvement of DM disease activity, a collective decision was made to continue chemotherapy and topical treatment without additional systemic intervention.

## Discussion

A myriad of epidemiological studies published over the last century established a strong association between DM and malignancy [1,2]. While adenocarcinoma is the most common type of cancer in DM, the risk for squamous cell and hematologic cancers also appears to be increased [5]. An analysis of hematologic malignancy in patients with polymyositis (PM) and DM identified B-cell lymphoma as the predominant lymphoproliferative disorder [6]. In the majority of these cases, PM/DM preceded hematologic malignancy onset - similar to what is seen in solid tumor literature [6]. MDS, however, has rarely been reported in connection with DM despite a known link between MDS and development of other autoimmune diseases like vasculitis and seronegative arthritis [7-10]. Palterer et al. recently described a patient with anti-TIF1gamma antibody-positive DM diagnosed with low-risk MDS after an extensive workup for malignancy. MDS was stable without excess blasts and required no therapy; importantly, the disease course of DM was not discussed [10]. Case reports of DM associated with MDS are summarized in Table 1.

**Table 1:** Summary of dermatomyositis (DM) cases associated with myelodysplastic syndrome (MDS) DM: dermatomyositis. MDS: myelodysplastic syndrome. Ref: reference. M: male. F: female. MRI: magnetic resonance imaging. CK: creatine kinase. LD: lactate dehydrogenase. ANA: antinuclear antibody. IVIG: intravenous immunoglobulin.

Ref.	Age/ Sex	Race	Dermatomyositis Onset	Cutaneous Features	Muscle Involvement	Laboratory Values	Autoantibodies	Treatment	Response
[8]	50/M	Unknown	3 months post MDS	Gottron's papules, heliotrope rash, periungual telangiectasia, shawl sign, V-sign	Concomitant with cutaneous eruption	Elevated CK, LD, and aldolase	Positive for ANA; Negative for anti-RNP, anti-Scl-70, anti-Jo-1	Systemic steroids, followed by methotrexate	DM resolution over 4 months
[9]	66/F	Japanese	1 month prior to MDS	Gottron's papules, shawl sign, V-sign	Concomitant with cutaneous eruption	Elevated LD and aldolase; Normal CK	Negative for ANA; Negative for anti-Jo-1	Systemic steroids, followed by cyclophosphamide	Refractory DM
[10]	78/F	Unknown	Immediately prior to MDS	Gottron's papules, heliotrope rash, periorbital edema, periungual telangiectasia, shawl sign, V-sign	Concomitant with cutaneous eruption	Normal CK and aldolase	Positive for ANA; Positive for anti-TIF1gamma	Systemic steroids, IVIG, followed by methotrexate	Cutaneous disease course unknown
Our Patient	70/M	Caucasian	Immediately prior to MDS	Red-on-white poikiloderma of scalp, heliotrope rash, periorbital edema, periungual telangiectasia, shawl sign, V-sign	MRI findings indicative of myopathy 6 months following cutaneous eruption	Minimally elevated aldolase; Normal CK	Negative for ANA; Positive for anti-TIF1gamma	Topical steroids; MDS chemotherapy (azacytidine and pevonedistat)	Cutaneous improvement over 10 weeks of MDS chemotherapy

Here we reported a case of DM that initially presented with severe cutaneous features in a patient with anti-TIF1gamma antibodies and subsequent diagnosis of MDS with EB-2. Two cycles of azacytidine and pevonedistat chemotherapy resulted in significant clinical and symptomatic improvement of cutaneous DM. Indeed, cancer-associated DM is thought to be driven by a paraneoplastic autoimmune response with tumor antigen cross-reactivity in normal muscle and skin [1]. This is supported by the high prevalence of autoantibodies in cancer-associated DM, including anti-TIF1gamma and anti-NXP-2. Anti-TIF1gamma antibody-positive DM usually presents with extensive cutaneous disease, but only mild muscle weakness; cutaneous findings more specific to anti-TIFgamma antibody-positive DM include red-on-white poikiloderma of the scalp and extensive psoriasiform lesions - features that were prominent in our patient (Figure 1) [2,11]. Recent data suggests that most patients who achieve a clinical response of malignancy also achieve a clinical response of DM [12]. Moreover, factors that positively correlate with clinical response of DM include increased age and malignancy [12]. Consequently, MDS chemotherapy was continued as planned, without additional interventions to specifically target myopathy. 

## Conclusions

In summary, this interesting case not only identifies a novel association between MDS and anti-TIF1gamma antibody-positive DM but also reinforces the idea that autoantibody testing and screening for malignancy should be considered in all patients presenting with DM, particularly in the elderly with severe or recalcitrant disease.
